# The Unusual Lipid A Structure and Immunoinhibitory Activity of LPS from Marine Bacteria *Echinicola pacifica* KMM 6172^T^ and *Echinicola vietnamensis* KMM 6221^T^

**DOI:** 10.3390/microorganisms9122552

**Published:** 2021-12-10

**Authors:** Molly Dorothy Pither, Giuseppe Mantova, Elena Scaglione, Chiara Pagliuca, Roberta Colicchio, Mariateresa Vitiello, Oleg V. Chernikov, Kuo-Feng Hua, Maxim S. Kokoulin, Alba Silipo, Paola Salvatore, Antonio Molinaro, Flaviana Di Lorenzo

**Affiliations:** 1 Department of Chemical Sciences, University of Naples Federico II, Via Cinthia 4, 80126 Naples, Italy; mollydorothy.pither@unina.it (M.D.P.); silipo@unina.it (A.S.); molinaro@unina.it (A.M.); 2 Department of Molecular Medicine and Medical Biotechnologies, University of Naples Federico II, Via S. Pansini n 5, 80131 Naples, Italy; Giuseppe.mantova@unina.it (G.M.); elena.scaglione@unina.it (E.S.); chiara.pagliuca@unina.it (C.P.); roberta.colicchio@unina.it (R.C.); mariateresa.vitiello2@unina.it (M.V.); psalvato@unina.it (P.S.); 3 Department of Chemical, Materials and Production Engineering, University of Naples Federico II, Piazzale V. Tecchio 80, 80125 Naples, Italy; 4 G.B. Elyakov Pacific Institute of Bioorganic Chemistry, Far Eastern Branch, Russian Academy of Sciences, 159/2, Prospect 100 Let Vladivostoku, 690022 Vladivostok, Russia; chernikov@piboc.dvo.ru (O.V.C.); maxchem@mail.ru (M.S.K.); 5 Department of Biotechnology and Animal Science, National Ilan University, No. 1, Sec. 1, Shen-Lung Road, Ilan 26099, Taiwan; kuofenghua@gmail.com; 6 Task Force on Microbiome Studies, University of Naples Federico II, 80126 Naples, Italy; 7 CEINGE-Biotecnologie Avanzate s.c.ar.l., Via G. Salvatore n 436, 80131 Naples, Italy; 8 Department of Agricultural Sciences, University of Naples Federico II, Via Università 100, 80055 Portici, Italy

**Keywords:** marine bacteria, *Echinicola*, lipopolysaccharide (LPS), lipid A, structural characterization, TLR4 antagonists

## Abstract

Gram-negative bacteria experiencing marine habitats are constantly exposed to stressful conditions dictating their survival and proliferation. In response to these selective pressures, marine microorganisms adapt their membrane system to ensure protection and dynamicity in order to face the highly mutable sea environments. As an integral part of the Gram-negative outer membrane, structural modifications are commonly observed in the lipopolysaccharide (LPS) molecule; these mainly involve its glycolipid portion, i.e., the lipid A, mostly with regard to fatty acid content, to counterbalance the alterations caused by chemical and physical agents. As a consequence, unusual structural chemical features are frequently encountered in the lipid A of marine bacteria. By a combination of data attained from chemical, MALDI-TOF mass spectrometry (MS), and MS/MS analyses, here, we describe the structural characterization of the lipid A isolated from two marine bacteria of the *Echinicola* genus, i.e., *E. pacifica* KMM 6172^T^ and *E. vietnamensis* KMM 6221^T^. This study showed for both strains a complex blend of mono-phosphorylated tri- and tetra-acylated lipid A species carrying an additional sugar moiety, a d-galacturonic acid, on the glucosamine backbone. The unusual chemical structures are reflected in a molecule that only scantly activates the immune response upon its binding to the LPS innate immunity receptor, the TLR4-MD-2 complex. Strikingly, both LPS potently inhibited the toxic effects of proinflammatory *Salmonella* LPS on human TLR4/MD-2.

## 1. Introduction

Marine environments host remarkably high and diverse microbial populations considered as the oldest life on the planet. These microorganisms have adapted, over millions of years, to survive and proliferate in seas and oceans, which are extraordinarily delicate and highly mutable, besides being often characterized by one or more extreme chemical or physical parameters (i.e., high salinity, low pressure, and low temperature) [1]. Unveiling the molecular mechanisms at the basis of the microbial adaptation to this complex environment is a prolific and intriguing area of research, with marine bacteria attracting most researchers’ attention. Indeed, marine bacteria represent a fascinating taxonomic lineage and a source of natural biologically active compounds comprising a broad range of antitoxins, antibiotics, antitumor, antimicrobial agents, and enzymes with a wide activity spectrum [2]. The cell envelope of marine bacteria is in constant contact with the stressors of the surrounding environment, which implies necessarily that its main constituents (membrane lipids, proteins, and glycoconjugates) must be precisely organized to maintain the proper physiology and functionality, even in the “extreme” aquatic habitat. At the same time, these membrane components must be versatile enough to face the constantly changing environment. 

Marine bacteria are predominantly Gram-negative; therefore, their cell envelope has a 3-layered architecture consisting of a cytoplasmic membrane, a periplasmic space containing the peptidoglycan layer and soluble proteins, and an outer membrane whose exterior leaflet is covered by lipopolysaccharides (LPS) [3,4,5]. LPS are typically made up of three different constituents: a glycolipid anchor to the outer membrane, the lipid A, an oligosaccharide (core OS), and a polysaccharide portion (O-chain), which is the outermost part of the whole molecule. An LPS expressing all three domains is denominated a smooth-type LPS (S-LPS); whereas, when the O-chain portion is missing it is named rough-type LPS (R-LPS or LOS) [4,5,6]. Due to their external location, LPS are involved in several biological activities ranging from membrane stabilization to the defense against dangerous outside agents [4,5,6]. Nevertheless, LPS are certainly known for their central role in many aspects of host–microbe interaction events, such as, among others, colonization, symbiosis, and virulence [3,4,5,6]. For bacteria that interact with humans, in fact, LPS is recognized by the innate immunity receptor complex built up of Toll-like receptor 4 (TLR4) and myeloid differentiation factor-2 (MD-2), which is located on several types of immune cells. Upon this recognition event the host innate immune response is triggered, with the consequent elicitation of an inflammatory response. Therefore, LPS from bacteria usually are seen as a danger for human health, leading, in certain cases, to dramatic outcomes such as sepsis and septic shock [7].

In this frame, the lipid A chemical structure is extremely important as it is directly correlated to the immunopotential of the majority of LPS isolated from pathogenic Gram-negative bacteria, being the part specifically recognized by the TLR4/MD-2 complex. Indeed, even the subtler chemical modification of the lipid A has repercussions on the capability of the entire LPS molecule to trigger potent, weak, or no immune response by host cells [3,4,5,6,7]. Therefore, depending on the structure of the lipid A, an LPS can behave as a strong or weak activator of the TLR4-mediated inflammatory response (defined as an agonist or a weak agonist). In contrast, some other LPS/lipid A structures result in TLR4 antagonists that are ineffective in activating the TLR4-mediated signaling while still keeping the ability to bind the receptor and to prevent the binding of other agonistic LPS; acting as such, antagonistic LPS/lipid A are able to limit the dangerous effects that are caused by potent inflammatory LPS on host cells [5,8,9]. This LPS structure-dependent capability to modulate the host immune response has boosted the interest of many researchers in the chemical, biomedical, and pharmacological fields attracted by the possibility to finely tune the immune response and, hence the inflammatory processes. In particular, LPS molecules and/or synthetic derivatives that are capable of suppressing or impeding the TLR4/MD-2 activation by competing with inflammatory LPS are considered attractive candidates for production of new therapeutics to combat sepsis [5,8,9].

In this frame, LPS expressing unusual structural features are considered as potential inhibitors of the TLR4-mediated signaling. Manifold studies have revealed peculiar and uncommon LPS chemical structures from marine bacteria, which were reflected in an interesting immunological behavior that comprises a very weak immunostimulant activity or even antagonistic properties [3,10,11,12]. Altogether, the structural elucidation of the lipid A from marine bacteria is certainly a fundamental starting point for the comprehension of the molecular details at the basis of adaptation processes, but it is also extremely important in the perspective of the realization of new generation immune-therapeutics inspired by a natural source. In light of this, we have investigated the *Echinicola* genus that belongs to the phylum Bacteroidetes and comprises Gram-negative pigmented bacteria of marine origin. The genus *Echinicola* accommodates only a few species that have been isolated from diverse marine sources and that are typically distinct from each other by several phenotypic features [13]. Here we report on two strains of this genus, *E. pacifica* KMM 6172^T^ isolated from the sea urchin *Strongylocentrotus intermedius* (Sea of Japan) [14], and *E. vietnamensis* KMM 6221^T^ isolated from seawater collected in a mussel farm located in a lagoon of Nha Trang Bay (South China Sea) [15]. Notably, we have previously shown that both bacteria express an S-LPS with unusual O-chain structures built up of a pentasaccharide repeating unit containing d-galactose, l-rhamnose, 2-acetoamido-2-deoxy-d-glucose, and 2,3-diacetamido-2,3-dideoxy-d-glucuronic acid for *E. pacifica* KMM 6172^T^ [14], and a tetrasaccharide composed of 2-acetamido-2-deoxy-d-glucuronic acid, 2-acetamido-2-deoxy-d-galactose, d-glucuronic acid, and 3,6-dideoxy-l-*xylo*-hexose for *E. vietnamensis* KMM 6221^T^ [15]. 

In this study, we describe the structural characterization of the lipid A moiety of both *Echinicola* strains by merging data attained from chemical analyses and from a Matrix-Assisted Laser Desorption/Ionization-Time of Flight Mass Spectrometry (MALDI-TOF MS) and tandem MS (MS/MS) study. Both these marine bacterial strains turned out to express highly heterogeneous mixtures of mono-phosphorylated hypo-acylated lipid A species characterized by the presence of d-galacturonic acid (d-GalA) as an additional sugar constituent of their lipid A saccharide backbone. Intrigued by the unusual chemical structures, we have also evaluated the immunoactivity of the isolated LPS that showed to act both as weak TLR4 agonists and as inhibitors of the inflammatory response triggered by inflammatory LPS on HEK-Blue^TM^ human (h) TLR4 cells.

## 2. Materials and Methods

### 2.1. Bacteria Isolation and Growth

*E. pacifica* type strain KMM 6172^T^ (=KCTC 12368^T^ = LMG 23350^T^) and *E*. *vietnamensis* type strain KMM 6221^T^ (=DSM 17526^T^ = LMG 23754^T^) were both from the Collection of Marine Microorganisms (KMM) of the G.B. Elykov Pacific Institute of Bioorganic Chemistry, Far Eastern Branch of Russian Academy of Sciences. Both strains were cultivated for 48 h at ambient temperature on a medium composed of (/L): 5.0 g Bacto peptone (Difco, Franklin Lakes, NJ, USA), 2 g Bacto yeast extract (Difco), 0.2 g KH_2_PO_4_, 1.0, g glucose, and 0.05 g MgSO_4_ in 50% (*v*/*v*) distilled water and 50% (*v*/*v*) natural seawater [14,15]. 

### 2.2. Isolation and Purification of the LPS from E. pacifica KMM 6172^T^ and E. vietnamensis KMM 6221^T^

Lyophilized bacterial cells were extracted following the hot phenol/water procedure [16], and the extracted material was extensively dialyzed against distilled water (Spectra/Por^®^, Fisher Sci. Leicestershire, UK, cut-off 12–14 kDa). The nature and degree of purity of the extracted LPS was screened by Sodium Dodecyl Sulphate-PolyAcrylamide Gel Electrophoresis (SDS-PAGE), followed by silver nitrate-gel staining [17]. To remove cell contaminants, such as proteins and nucleic acids, an enzymatic digestion with RNase (Sigma–Aldrich, Darmstadt, Germany), DNase (Sigma–Aldrich, Darmstadt, Germany), and protease (Sigma–Aldrich, Darmstadt, Germany) (37 and 56 °C) was performed, followed by an exhaustive dialysis against distilled water (Spectra/Por^®^, Fisher Sci., Leicestershire, UK, cut-off 12–14 kDa). An ultracentrifugation step (200,000× *g*, 4 °C, 16 h) and a size-exclusion chromatography on a Sephacryl high-resolution S-500 column (GE Healthcare, Buckinghamshire, UK) were also performed as additional steps of purification for both the extracted LPS. The fractions eluted by the column were checked by SDS-PAGE and those containing LPS were carried forward for further analysis. Finally, in order to remove any phospholipids possibly contaminating the isolated LPS, several washes with a mixture of CHCl_3_/CH_3_OH (1:2, *v*/*v*) and CHCl_3_/CH_3_OH/H_2_O (3:2:0.25, *v*/*v*) were executed. After the removal of organic solvents, the LPS were lyophilized and then underwent a set of compositional analyses.

### 2.3. Fatty Acid Compositional Analysis of LPS from E. pacifica KMM 6172^T^ and E. vietnamensis KMM 6221^T^

The total fatty acid content was ascertained by treating each LPS with 4 M HCl (100 °C, 4 h), followed by a treatment with 5 M NaOH (100 °C, 30 min). Fatty acids were then extracted in chloroform, after the adjustment of the pH, and then methylated with diazomethane, and analyzed by Gas Chromatography Mass Spectrometry (GC-MS). The ester-bound fatty acids were released after treatment with aqueous 0.5 M NaOH in CH_3_OH (1:1, *v*/*v*, 85 °C, 2 h), followed by acidification of the products, extraction in chloroform and methylation with diazomethane. The obtained fatty acids were then analyzed via GC-MS. An aliquot of each LPS fraction was also treated with 1.25 M HCl/CH_3_OH (85 °C, 16 h). The mixture was extracted with hexane that contained the fatty acids as methyl ester derivatives, which were then injected and inspected by GC-MS. To determine the absolute configuration of the 3-hydroxy fatty acids, these were released through a treatment with 4 M NaOH (100 °C, 5 h) and then converted into 3-methoxy acid l-phenylethylamides and analyzed via GC-MS [18]. By comparing the retention times of authentic l-phenylethylamides of various standard fatty acids with those derived from the examined LPS, it was possible to assign the (*R*) configuration to all the hydroxylated fatty acids identified for the two marine bacteria lipid A. The analyses were all executed on an Agilent Technologies gas chromatograph 6850A equipped with a mass selective detector 5973N and a Zebron ZB-5 capillary column (Phenomenex, Torrance, CA, USA, 30 m × 0.25 mm internal diameter, flow rate 1 mL/min, He as carrier gas). The following temperature program was employed for the lipid analysis: 140 °C for 5 min, 140 °C→280 °C at 10 °C/min, and 280 °C for 10 min.

### 2.4. Isolation and Analysis of the Lipid A Fractions

An aliquot of each LPS (15 mg) underwent a mild acid hydrolysis with acetate buffer (pH 4.4, 2 h, 100 °C). A mixture of CH_3_OH and CHCl_3_ was added to the acid hydrolysis product to obtain a CH_3_OH/CHCl_3_/hydrolysate 2:2:1.8 (*v*/*v*/*v*) ratio. This mixture was then shaken and centrifuged (4 °C, 8000× *g*, 20 min). The chloroform phase, containing the lipid A, was collected and washed with the water phase of a freshly prepared Bligh/Dyer mixture (CHCl_3_/CH_3_OH/H_2_O, 2:2:1.8) [19]. The organic phases were pooled, dried, and inspected by MALDI-TOF MS. In parallel, in order to gain information about the nature of the lipid A sugar backbone, an aliquot of each lipid A fraction underwent methanolysis (1.25 M HCl/CH_3_OH, 85 °C, 16 h) followed by an acetylation step (80 °C, 20 min) and GC-MS analysis [20]. The temperature program in this case was: 150 °C for 5 min, 150 °C→ 280 °C at 3 °C/min, and 280 °C for 5 min. The d-configuration of the glucosamine and the galacturonic acid constituting the lipid A saccharide backbone has been defined by GC-MS analysis of the acetylated *O*-(+)-2-octyl glycoside derivatives and comparison with authentic standards [21]. Finally, to unequivocally establish the location of the fatty acids with respect to the glucosamine disaccharide backbone, an aliquot of lipid A fraction (0.5 mg) was treated with ammonium hydroxide (NH_4_OH) as previously reported [22]. The sample was then dried, suspended in distilled water, lyophilized, and then analyzed by MALDI-TOF MS along with the untreated lipid A fraction.

### 2.5. MALDI-TOF Mass Spectrometry Analysis

MS and the MS/MS experiments were performed both in linear and reflectron mode, negative-ion polarity on an ABSCIEX TOF/TOF 5800 Applied Biosystems (Foster City, CA, USA) mass spectrometer equipped with an Nd:YAG laser (λ = 349 nm), with a 3 ns pulse width and a repetition rate of up to 1000 Hz, and also equipped with delayed extraction technology. Lipid A fractions obtained after mild acid hydrolysis of each LPS were dissolved in CHCl_3_/CH_3_OH (50:50, *v*/*v*). The matrix solution in this case was 2,4,6-trihydroxyacetophenone (THAP) dissolved in CH_3_OH/0.1% trifluoroacetic acid/CH_3_CN (7:2:1, *v*/*v*/*v*) at a concentration of 75 mg/mL [20,23]. The NH_4_OH-treated lipid A fractions were instead dissolved in CHCl_3_-trifluoroethanol (4:1, *v*/*v*) and the matrix used was 2,5-dihydroxybenzoic acid (DHB) (Sigma Aldrich^®^) in acetonitrile 0.2% trifluoroacetic acid (7:3, *v*/*v*) [20,22]. To analyze the bacterial pellet, the matrix used was DHB (10 mg/mL) in CHCl_3_/CH_3_OH (9:1, *v*/*v*) [24]. In all cases, 0.5 μL of the sample and 0.5 μL of the matrix solution were deposited onto a stainless-steel plate and left to dry at room temperature. For MS experiments each spectrum was a result of the accumulation of 2000 laser shots, whereas 5000–7000 shots were summed for the MS/MS spectra. Each experiment was performed in triplicate.

### 2.6. HEK Cell Cultures

HEK-Blue^TM^ hTLR4, HEK-Blue^TM^ hTLR2, and HEK-Blue^TM^ Null2^TM^ cells (InvivoGen, San Diego, CA, USA) were cultured at 37 °C in 5% CO_2_ using Dulbecco’s minimal essential media (DMEM) supplemented with 10% heat-inactivated fetal bovine serum (FBS) (Microgem, Naples, Italy), 1% glutamine (Himedia, Einhausen, Germany), 1% penicillin/streptomycin (Himedia, Einhausen, Germany), and 100 µg/mL Normocin (InvivoGen). Selection of the plasmids in HEK-Blue^TM^ hTLR4 and hTLR2 cells required the use of a mixture of selective antibiotics (HEK-Blue^TM^ Selection) (InvivoGen), and in HEK-Blue Null2^TM^ cells required the use of 100 µg/mL Zeocin (InvivoGen).

### 2.7. HEK-Blue^TM^ Null2, hTLR2 and hTLR4 Stimulation and Competition Assays

HEK-Blue^TM^ hTLR4 cells were seeded into 96-well plates (3 × 10^5^ cells per well) and incubated with different concentrations of *E. pacifica* KMM 6172^T^ LPS, *E. vietnamensis* KMM 6221^T^ LPS, or *Salmonella typhimurium* SH 2201 LPS (1, 10, 100 ng/mL) for 18 h to analyze Nuclear Factor kappa B (NF-κB) activation by evaluating NF-κB-dependent secreted alkaline phosphatase (SEAP) using QUANTI-Blue^TM^ (InvivoGen). After this time, the supernatants were collected, and Interleukin 8 (IL-8) release was measured through ELISA (Invitrogen, Waltham, MA, USA). HEK-Blue^TM^ hTLR2 and HEK-Blue^TM^ Null2^TM^ cell lines were exposed to *E. pacifica* KMM 6172^T^ LPS, *E. vietnamensis* KMM 6221^T^ LPS, or *S. typhimurium* SH 2201 LPS (1, 10, 100 ng/mL) for 18 h, and for HEK-Blue^TM^ hTLR2 cells also to Pam3CSK4 (500 ng/mL) (InvivoGen) and analyzed as above. For the competition assays, HEK-Blue^TM^ hTLR4 cells were pretreated with *E. pacifica* KMM 6172^T^ LPS or *E. vietnamensis* KMM 6221^T^ LPS (1, 10 and 100 ng/mL) for 90 min and then stimulated with *S. typhimurium* SH 2201 LPS (1 ng/mL) for 18 h. After this time, the NF-κB activity and IL-8 release were measured as described above.

## 3. Results

### 3.1. Isolation and Fatty Acid Compositional Analysis of the LPS from E. pacifica KMM 6172^T^ and E. vietnamensis KMM 6221^T^

LPS from bacterial cells of *E. pacifica* KMM 6172^T^ and *E. vietnamensis* KMM 6221^T^ were isolated through the hot phenol/water method [16]. The nature of the extracted material was established by SDS-PAGE after silver-nitrate gel staining [17]. The gel showed, for both strains, a ladder-like pattern typical of an O-chain-containing LPS (i.e., an S-LPS) (Figure S1a), which was in agreement with our previous analysis [14,15]. The extracted LPS underwent extensive steps of purification comprising enzymatic treatment, dialysis, ultracentrifugation, and size-exclusion chromatography. 

After purification, a detailed compositional analysis was performed on both LPS to establish the fatty acid content (results are summarized in Table 1). This analysis highlighted an extremely heterogenous composition, which was however qualitatively equal for the two *Echinicola* strains. 

In order to define the structure of the lipid A moiety of both the *Echinicola* strains, an aliquot of each purified LPS was subjected to a mild acid hydrolysis in order to selectively cleave the acid labile linkage between the Kdo, the first monosaccharide of the core OS, and the nonreducing glucosamine of the lipid A portion. After purification through Bligh–Dyer extraction [19], an aliquot of the isolated lipid A fractions underwent a detailed MALDI-TOF MS and MS/MS investigation, while another aliquot was subjected to methanolysis followed by an acetylation step in order to analyze the fatty acids as methyl esters derivatives and to define the nature of the lipid A saccharide backbone. This analysis confirmed the fatty acid content reported in Table 1, and showed the occurrence, as expected, of the acetylated methyl glycoside derivative of 2-amino-2-deoxy-d-glucose (d-glucosamine), but also it revealed the occurrence of d-GalA (Figure S1b,c), thus, suggesting the presence of an additional decoration of the lipid A of both *Echinicola* strains by this acid sugar residue.

### 3.2. MALDI-TOF MS and MS/MS Analysis on the Isolated Lipid A from E. pacifica KMM 6172^T^

**Figure 1:**
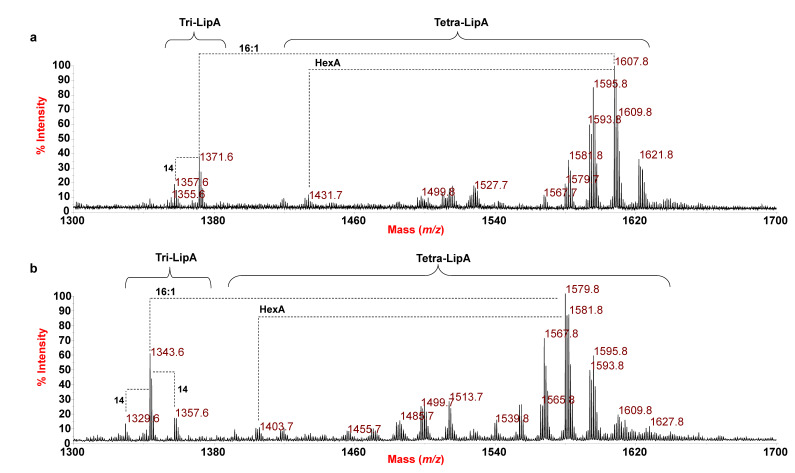
Reflectron MALDI-TOF mass spectra, recorded in negative polarity, of lipid A from *E. pacifica* KMM 6172^T^ (**a**) and *E. vietnamensis* KMM 6221^T^ (**b**) obtained after acetate buffer treatment of each LPS. The lipid A species are labelled as Tri- and Tetra-Lip A indicating the degree of acylation. “HexA” indicates the hexuronic acid (i.e., the d-GalA).

The reflectron MALDI-TOF mass spectrum, recorded in negative-ion polarity, of the lipid A from *E. pacifica* KMM 6172^T^ is reported in Figure 1a. Two main clusters of peaks were identified and matched with deprotonated [M-H]^−^ tri-acylated (*m*/*z* 1343.6–1371.6) and tetra-acylated lipid A species (*m*/*z* 1499.8–1635.8). The extreme complexity and heterogeneity of the tetra-acylated lipid A species was immediately apparent as proven by the occurrence of peaks differing in 14 (–CH_2_– unit), 28 amu (–CH_2_CH_2_– unit), and 2 amu, which is typically diagnostic for the presence of lipid A species that diverge in the length of their acyl moieties as well as for the occurrence of unsaturated fatty acids, which was in agreement with compositional analysis. Moreover, the spectrum showed additional peaks in the *m*/*z* range 1417.7–1445.7 that differ in 176 amu from the main tetra-acylated lipid A species, which likely suggested the occurrence of lipid A forms characterized by the presence of a hexuronic acid (HexA) modification on the lipid A saccharide backbone, also in accordance with chemical analyses. 

In detail, in the mass region *m*/*z* 1499.8–1635.8 (Figure 1a and Table 2), mono-phosphorylated tetra-acylated lipid A species decorated by one HexA unit were identified. The main peak at *m*/*z* 1607.8 was assigned to a tetra-acylated lipid A species composed of the typical glucosamine disaccharide backbone substituted by one phosphate and one HexA unit, bearing two *i*17:0(3-OH), one 15:0(3-OH) [or *i*15:0(3-OH)], and one 16:1; whereas the peak at *m*/*z* 1527.7 matched with a mono-phosphorylated lipid A species, decorated by HexA, carrying two *i*17:0(3-OH), one 15:0(3-OH) [or *i*15:0(3-OH)], and one *i*9:0(3-OH). Finally, in the mass region *m*/*z* 1343.6–1371.6, tri-acylated lipid A species carrying one phosphate and one HexA unit were also identified, with the main peak at *m*/*z* 1371.6 identified as a lipid A species carrying two *i*17:0(3-OH) and one 15:0(3-OH) [or *i*15:0(3-OH)]. For description purposes, hereafter the hydroxylated 15:0 acyl moiety will be mentioned simply as 15:0(3-OH), taking into consideration that it can be also found in its iso-branched structure, according to compositional analysis (Table 1). To exclude that such a hypo-acylated lipid A could derive from the mild acid treatment executed on the LPS, we also performed a MALDI-TOF MS analysis directly on the bacterial pellet following the Larrouy-Maumus et al. (2016) protocol [24]. Interestingly, the negative-ion MALDI-TOF mass spectrum (Figure S2a) confirmed the structural elucidation deduced by analysis of the isolated lipid A fraction (Figure 1a), thus, excluding any lack of structural information possibly occurring as a consequence of the chemical treatment to isolate the lipid A.

**Figure 2:**
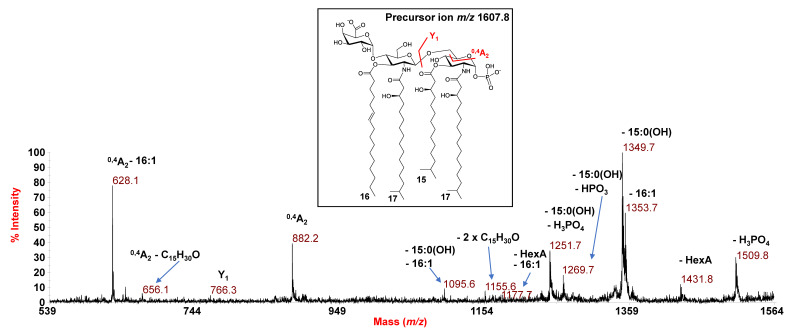
Negative-ion MALDI MS/MS spectrum of precursor ion at *m*/*z* 1607.8, a representative ion peak of the cluster ascribed to tetra-acylated lipid A species from *E. pacifica* KMM 6172^T^. The assignment of main fragments is reported in the spectrum. The proposed structure for the lipid A species is reported in the inset, where the representation of 15:0(3-OH) in its branching form is tentative. In addition, since both the phosphate group and the HexA unit may be candidates to hold the charge, the proposed structure has been sketched carrying two negative charges for information purposes only. Peaks originating from the loss of C_15_H_30_O (226 mass units), due to the rearrangement occurring on amide-linked 3-OH acyl chains having the hydroxyl group free, have been also indicated.

A negative-ion MS/MS investigation was conducted to unveil the exact location of the lipid A acyl moieties with respect to the glucosamine disaccharide backbone as well as the position of the phosphate and the HexA residue. In detail, the MS/MS spectrum of the precursor ion at *m*/z 1607.8 (Figure 2) showed two intense peaks at *m*/*z* 1349.7 and 1353.7 attributed to ions derived from the loss of a 15:0(3-OH) and a 16:1 fatty acid, respectively. Less intense peaks were observed at *m*/*z* 1509.8 and *m*/*z* 1431.8 and were assigned to fragments that originated from the loss of the phosphate group and the HexA unit, respectively (Figure 2). The peak at *m*/*z* 1251.7 was attributed to a fragment devoid of the phosphate group and the 15:0(3-OH) unit; whereas, an ion originated from the loss of the HexA unit and the 16:1 was identified at *m*/*z* 1177.7. The observation of the ion peak at *m*/*z* 1095.6, matching with a fragment caused by the sequential loss of one 15:0(3-OH) and one 16:1, was important for the structural characterization. Indeed, the presence of this peak gave a first indication that the unsaturated acyl moiety (16:1) was not bound as a secondary substituent of the primary ester linked 15:0(3-OH). In parallel, the occurrence of the ion at *m*/*z* 882.2, originating from the sugar ring fragmentation (^0,4^A_2_) [25], was fundamental to define the nature of the fatty acids that decorated the nonreducing glucosamine (namely, one *i*17:0(3-OH) and one 16:1) as well as the location of the HexA on such a glucosamine unit. In support of this hypothesis, an ion originated from the sugar ring fragmentation ^0,4^A_2_ plus the loss of the 16:1 acyl moiety was also assigned to the peak at *m*/*z* 628.1. Therefore, three main observations were key to locate the 16:1 as a primary ester-bound fatty acid of the nonreducing glucosamine (as sketched in the inset of Figure 2): (i) the presence of several peaks relative to fragments originated from the loss of the 16:1 moiety; (ii) the fact that, compared to their acyl and acyloxyacyl counterparts, the loss of secondary acyl substituents of primary amide-linked fatty acids are less commonly observed in MS/MS investigation; (iii) the absence of any peak indicative of the loss of a whole unit of a hydroxylated fatty acid bearing 16:1 as a secondary acyl substituent. This structural hypothesis was then definitively corroborated by the observation of the peak at *m*/*z* 1155.6 assigned to a fragment originating from a rearrangement occurring only if the *N*-linked fatty acids at positions C-2 and C-2’ have a free 3-OH group, i.e., they do not carry secondary acyl substituents. 

Indeed, an enamine to imine tautomerization followed by a six-membered ring-based rearrangement justifies the generation of the ion peak at *m*/*z* 1155.6, which derives from the loss of a C_15_H_30_O neutral fragment (226 amu) from each primary *N*-linked acyl chain possessing a free 3-OH group. Since such a rearrangement can occur only if no secondary acyl substituents are linked to position 3 of the amide-linked acyl chains [26], the occurrence of this peak unequivocally located the 16:1 as a primary ester-linked moiety. Finally, the location of the phosphate group was defined based on the detection of the Y_1_ ion (*m*/*z* 766.3) [25], originated from the cleavage of the glycosidic linkage of the glucosamine backbone, which in turn also confirmed the location on the reducing glucosamine unit of a *i*17:0(3-OH) and a 15:0(3-OH). Likewise, the structure of the lipid A species detected at *m*/*z* 1595.8 and 1621.8 were also elucidated (Figures S3 and S4), revealing a tetra-acylated lipid A carrying two *i*17:0(3-OH), one 15:0(3-OH), and one 15:0 for the species detected at *m*/*z* 1595.8, and a tetra-acylated species bearing two *i*17:0(3-OH), one 15:0(3-OH), and one 17:1 for the species at *m*/*z* 1621.8; in both cases, a phosphate decorates the reducing glucosamine and a HexA decorates the nonreducing glucosamine.

**Figure 3:**
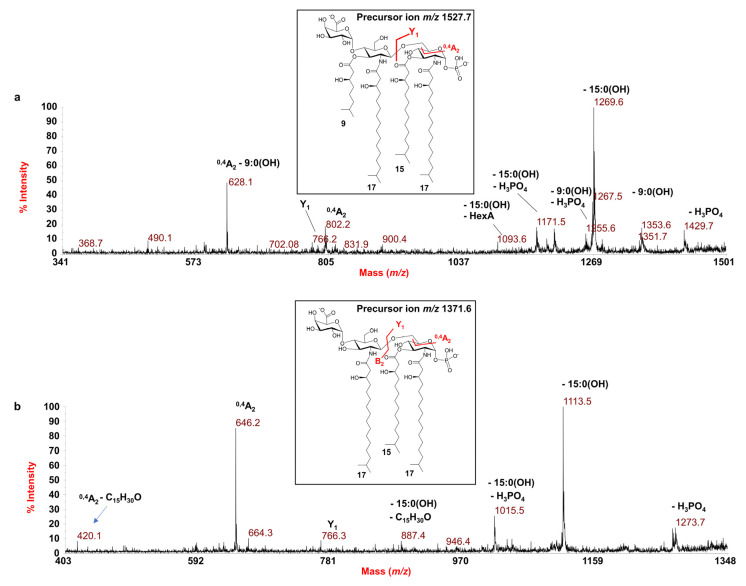
MALDI MS/MS analysis of tetra- (**a**) and tri-acylated (**b**) lipid A species from *E. pacifica* KMM 6172^T^. (**a**) Negative-ion MALDI MS/MS spectrum of precursor ion at *m*/*z* 1527.7, another representative ion peak of the cluster assigned to the tetra-acylated lipid A species. (**b**) Negative-ion MALDI MS/MS spectrum of precursor ion at *m*/*z* 1371.6, chosen as a representative of the cluster ascribed to the tri-acylated lipid A species. The assignment of the main fragments is indicated in the spectra. The proposed structure for the lipid A species is reported in each inset. The representation of 15:0(3-OH) is tentatively given in its branching form. As both the phosphate group and the HexA unit may hold the charge, the proposed structures have been sketched carrying two negative charges for information purposes only. Peaks originating from the loss of C_15_H_30_O (226 mass units) have been also indicated.

In order to further dissect the structure of such a heterogenous lipid A, the precursor ion at *m*/*z* 1527.7 was also chosen for negative-ion MS/MS analysis. The MS/MS spectrum (Figure 3a) showed an intense peak at *m*/*z* 1269.6 indicative of a fragment derived from the loss of a 15:0(3-OH) moiety, whose relative fragment also lacking the phosphate group was detected at *m*/*z* 1171.6. The peak detected at *m*/*z* 1353.6 was attributed to an ion originated from the loss of a *i*9:0(3-OH) moiety. Moreover, in this case, the cross-ring fragmentations ^0,4^A_2_ (*m*/*z* 802.2) and ^0,4^A_2_ minus a *i*9:0(3-OH) (*m*/*z* 628.1) were crucial to define the location and the nature of the acyl chains [namely one *i*17:0(3-OH) and one *i*9:0(3-OH)] on the nonreducing glucosamine as well as the position of the HexA unit. 

Finally, the investigation of the negative-ion MS/MS spectrum of the precursor ion at *m*/*z* 1371.6 (Figure 3b), chosen as a representative of the tri-acylated lipid A species, disclosed a lipid A composed of two *N*-linked *i*17:0(3-OH) and the *O*-linked 15:0(3-OH) acyl chains on a backbone made up of the glucosamine disaccharide substituted by HexA and one phosphate unit. This was suggested by the detection of the peak at *m*/*z* 646.2, derived from the cross-ring fragmentation ^0,4^A_2_, as well as of the Y_1_ ion peak at *m*/*z* 766.3 (Figure 3b), derived from the cleavage of the glycosidic bond.

**Figure 4:**
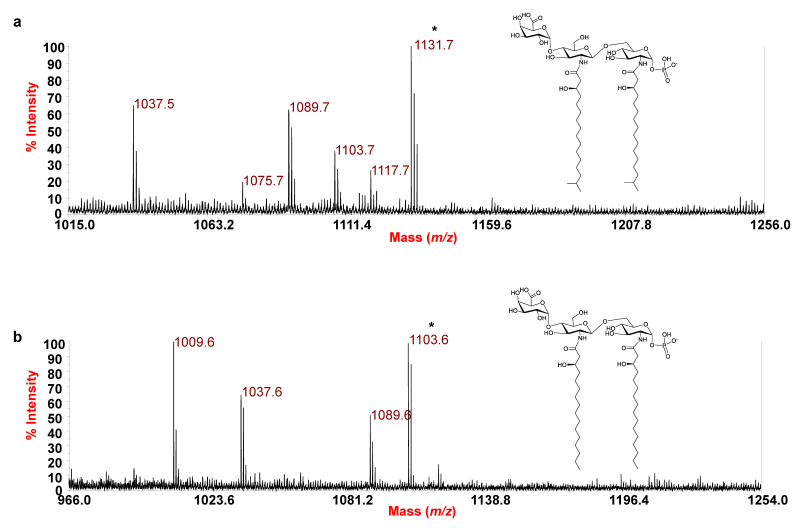
Reflectron MALDI-TOF mass spectrum, recorded in negative polarity, of lipid A from *E. pacifica* KMM 6172^T^ (**a**) and *E. vietnamensis* KMM 6221^T^ (**b**), after treatment with NH_4_OH. * Indicates the ion peak whose proposed structure is sketched in the inset, where the negative charge held by the phosphate is tentative.

In conclusion, in order to unequivocally establish the location of the acyl chains, an aliquot of lipid A was treated with NH_4_OH to selectively remove the acyl and acyloxyacyl esters, while leaving the acyl and acyloxyacyl amides unaltered. The negative-ion MALDI-TOF MS spectrum of the NH_4_OH-treated lipid A is reported in Figure 4a and shows a main peak at *m*/*z* 1131.7 matching with a lipid A carrying two *N*-linked *i*17:0(3-OH) besides being decorated by one phosphate and the HexA residue. Lipid A forms differing in 14 amu (-CH_2_-) were also identified. This analysis definitively confirmed that no secondary acyl substitutions occur in the *E. pacifica* KMM 6172^T^ lipid A. Thus, combining data from the MALDI-TOF MS and MS/MS analysis of the mild acid hydrolysis product with information attained from compositional analyses of both the whole LPS and the isolated lipid A fraction, it was possible to establish that the LPS from *E. pacifica* KMM 6172^T^ is made up of a complex blend of mono-phosphorylated tri- and tetra-acylated lipid A species mostly decorated by one phosphate on the reducing glucosamine unit and one D-GalA on the nonreducing glucosamine unit.

### 3.3. MALDI MS and MS/MS Analysis on the Isolated Lipid A from E. vietnamensis KMM 6221^T^

By an identical MS and MS/MS approach, the structure of the lipid A from *E. vietnamensis* KMM 6221^T^ was also established. As shown in Figure 1b, the negative-ion MS spectrum of the mild acid hydrolysis product and of the intact cells (Figure S2b) displayed a similar pattern of peaks to that observed for *E. pacifica* KMM 6172^T^, which, also in this case, was matched with a heterogenous mixture of mono-phosphorylated tri- and tetra-acylated lipid A species carrying the HexA decoration. However, differences in the predominant species between the two strains were observed. For example, the main peak at *m*/*z* 1579.8 was attributed to a tetra-acylated lipid A species carrying 16:0(3-OH), as primary *N*-linked fatty acids, and 15:0(3-OH) and 16:1 as the *O*-linked acyl chains. In relation to this peak, a tetra-acylated lipid A species devoid of the HexA was attributed to peak at *m*/*z* 1403.7; whereas, a tri-acylated lipid A form lacking the 16:1 unit was assigned to peak at *m*/*z* 1343.6. 

**Figure 5:**
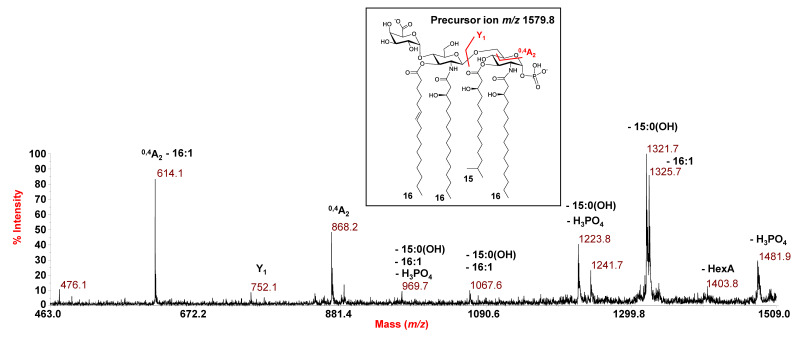
Negative-ion MALDI MS/MS spectrum of the precursor ion at *m*/*z* 1579.8, a representative ion peak of the cluster matching with tetra-acylated lipid A species from *E. vietnamensis* KMM 6221^T^. The assignment of main fragments is indicated. The proposed structure for the lipid A species is reported in the inset. In this case, the representation of 15:0(3-OH) in its branching form is tentative. Since both the phosphate group and the HexA unit may be candidates to hold the charge, the proposed structure has been depicted carrying two negative charges for information purposes only.

**Figure 6:**
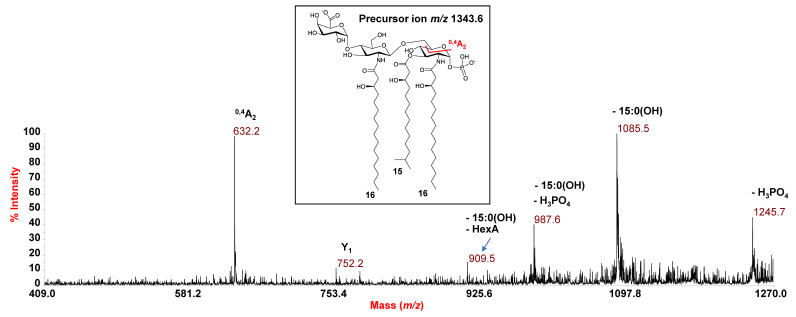
Negative-ion MALDI MS/MS spectrum of precursor ion at *m*/*z* 1343.6, a representative ion peak of the cluster ascribed to tri-acylated lipid A species from *E. vietnamensis* KMM 6221^T^. The assignment of fragments is reported. The proposed structure for the lipid A species is sketched in the inset carrying two negative charges for information purposes only in order to underline that both sites of the molecule (i.e., the phosphate and the GalA) may hold the negative charge.

Similar to *E. pacifica* KMM 6172^T^, a combination of data from negative-ion MS/MS analysis and MS investigation of the NH_4_-treated product enabled establishing the structure of the lipid A from *E. vietnamensis* KMM 6221^T^. Briefly, in the MS/MS spectrum of the precursor ion at *m*/*z* 1579.8 (Figure 5), no ions originating from the loss of a whole unit comprising a hydroxylated acyl moiety carrying a secondary fatty acid were detected. This, together with the observation of peaks matching with ions derived from the sequential loss of a 15:0(3-OH) and a 16:1, suggested that these acyl moieties were both *O*-linked to the glucosamine backbone, in addition to the amide-linked 16:0(3-OH). Nevertheless, more information on this aspect was gained by analysis of the MS/MS spectrum of the precursor ion at *m*/*z* 1513.7 (Figure S5), assigned to a tetra-acylated lipid A species carrying two *N*-linked 16:0(3-OH), and *O*-linked 10:0(3-OH) and 15:0(3-OH). Indeed, the occurrence of the peak at *m*/*z* 1089.7, deriving from the loss of 424 amu from the precursor ion, matched with an ion that lost a C_14_H_28_O neutral fragment (212 amu) from each primary *N*-linked acyl chain with a free 3-OH group. This corroborated the hypothesis of the absence of any secondary acyl substitution on the amide-linked fatty acids, which was definitively confirmed by analyzing the MS spectrum recorded on the lipid A product after NH_4_OH treatment (Figure 4b). The spectrum, in fact, showed a main species at *m*/*z* 1103.6 matching with a lipid A carrying the phosphate, the HexA and only the two *N*-linked 16:0(3-OH) acyl moieties. In conclusion, the MS/MS analysis conducted on the precursor ion at *m*/*z* 1343.6 (Figure 6) demonstrated that this mono-phosphorylated and tri-acylated species was composed of the two *N*-linked 16:0(3-OH) and the *O*-linked 15:0(3-OH) on the reducing glucosamine, as proven by the observation of the Y_1_ ion at *m*/*z* 752.2 and the ^0,4^A_2_ ion at *m*/*z* 632.2.

Therefore, as described for *E. pacifica* KMM 6172^T^, *E. vietnamensis* KMM 6221^T^ was shown to also produce an LPS with a remarkably heterogenous hypo-acylated lipid A carrying d-GalA and phosphate on the sugar backbone.

### 3.4. Immunological Properties of LPS from E. pacifica KMM 6172^T^ and E. vietnamensis KMM 6221^T^

**Figure 7:**
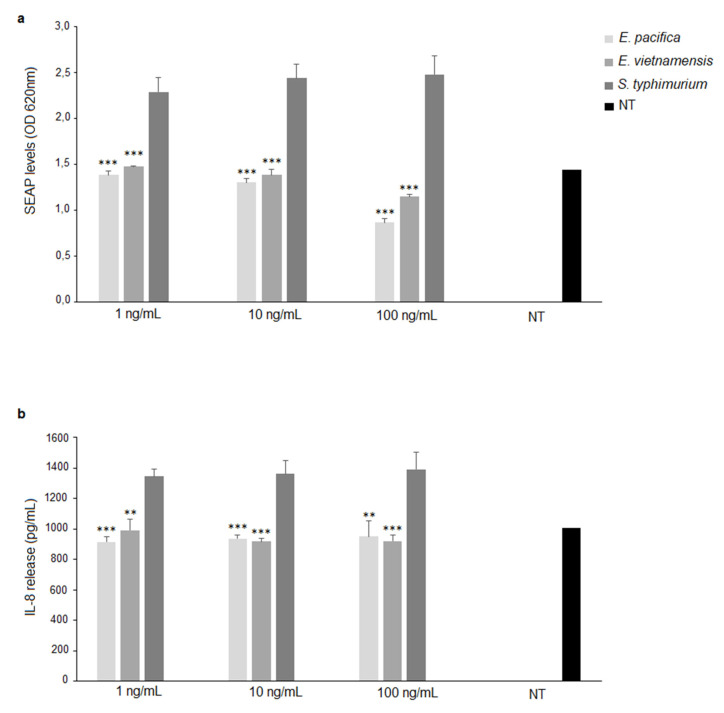
Stimulation of HEK Blue^TM^ hTLR4 cells. SEAP levels (OD) (**a**) and IL-8 release (pg/mL) (**b**) upon stimulation with LPS of *E. pacifica* KMM 6172^T^ (1, 10, 100 ng/mL) or *E. vietnamensis* KMM 6221^T^ LPS (1, 10, and 100 ng/mL); *S. typhimurium* SH 2201 LPS was used as positive control. Significant differences between *E. pacifica* KMM 6172^T^ LPS or *E. vietnamensis* KMM 6221^T^ LPS and *S. typhimurium* LPS values are indicated. ** *p* < 0.01, *** *p* < 0.001 by the Student *t*-test. NT, not treated cells. Data are expressed as mean ± SD of three independent experiments in triplicate.

The immunological activity of LPS from the two *Echinicola* strains was assessed in vitro in the model of HEK-Blue^TM^ hTLR4 cells, which are HEK293 cells stably transfected with human TLR4, MD-2, and CD14 genes. Activation of NF-κB was the read-out of this experiment. This was possible as HEK-Blue^TM^ hTLR4 cells also stably express a secreted embryonic alkaline phosphatase (SEAP) that is produced upon activation of NF-κB. Therefore, stimulation with a TLR4 ligand activates NF-κB and thereby the production of SEAP, which in turn is rapidly detected in cell culture media by an alkaline phosphatase substrate. Separately, we also assessed TLR4 involvement by measuring its downstream induction of IL-8 cytokine production in the same cell lines. HEK-Blue hTLR4 cells were stimulated with different concentrations (1, 10, and 100 ng/mL) of *E. pacifica* KMM 6172^T^ LPS or *E. vietnamensis* KMM 6221^T^ LPS. *S. typhimurium* SH 2201 LPS, containing fully hexa- and hepta-acylated lipid A, was used at the same concentrations as above, and evaluated as a positive control. Untreated cells were used as a negative control. As shown in Figure 7, both LPS from *Echinicola* strains induced a significantly lower NF-κB activation compared to cells treated with *S. typhimurium* LPS (*E. pacifica* KMM 6172^T^ LPS vs. *S. typhimurium* LPS, *p* < 0.001 at 1 ng/mL, 10 ng/mL, and 100 ng/mL, Figure 7a; *E. vietnamensis* KMM 6221^T^ LPS vs. *S. typhimurium* LPS, *p* < 0.001 at 1 ng/mL, 10 ng/mL, and 100 ng/mL, Figure 7a). In accordance with this outcome, IL-8 secretion was also lower after stimulation with both *Echinicola* LPS than after treatment with the LPS from *S. typhimurium* (*E. pacifica* KMM 6172^T^ LPS vs. *S. typhimurium* LPS, *p* < 0.001 at 1 ng/mL and 10 ng/mL; *p* < 0.01 at 100 ng/mL, Figure 7b; *E. vietnamensis* KMM 6221^T^ LPS vs. *S. typhimurium* LPS, *p* < 0.01 at 1 ng/mL; *p* < 0.001 at 10 ng/mL and 100 ng/mL Figure 7b). In addition, HEK-Blue^TM^ Null2^TM^ cells were used to evaluate the effective immunoactivation of *Echinicola* LPS through TLR4. The lack of any NF-kB activation and IL-8 production in HEK-Blue Null2 cells, i.e., cells without TLR4/MD-2/CD14 expression (Figure S6), indicated that NF-κB activation and IL-8 release by *E. pacifica* KMM 6172^T^ LPS and *E. vietnamensis* KMM 6221^T^ LPS were TLR4-dependent.

**Figure 8:**
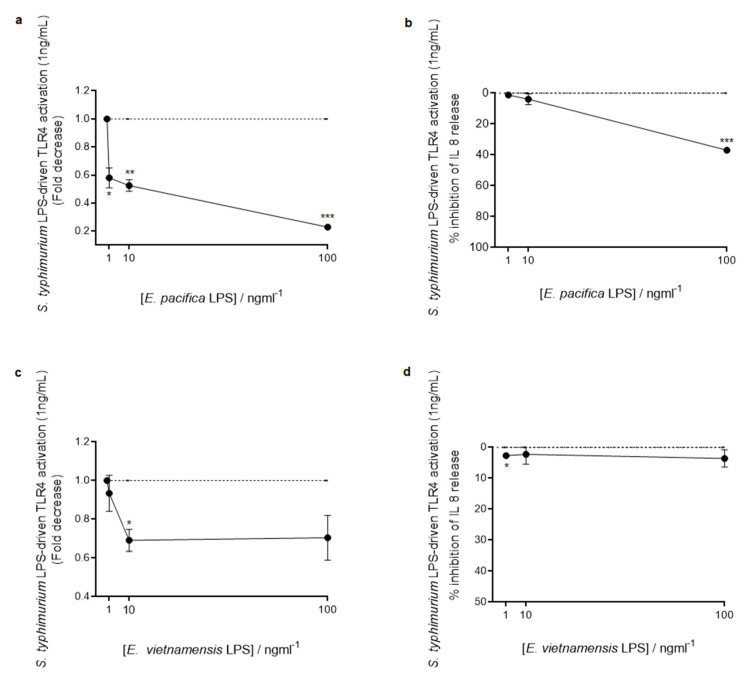
Competition assay. Fold decrease in NF-κB (**a**,**c**) and inhibition percentage of IL-8 release (**b**,**d**) in HEK Blue^TM^ hTLR4 cells pretreated with different concentrations (1, 10, and 100 ng/mL) of *E. pacifica* KMM 6172^T^ LPS (**a**,**b**) or *E. vietnamensis* KMM 6221^T^ LPS (**c**,**d**) and then stimulated with *S. typhimurium* LPS (1 ng/mL). Significant differences between the pretreatment with *E. pacifica* KMM 6172^T^ LPS or *E. vietnamensis* KMM 6221^T^ LPS and *S. typhimurium* LPS alone values are indicated. * *p* < 0.05, ** *p* < 0.01 and *** *p* < 0.001 by the Student’s *t*-test.

Since some LPS isolated from marine bacteria have shown a fascinating potential as inhibitors of the proinflammatory action of LPS from pathogenic bacteria, we also investigated the capability of LPS from the two *Echinicola* strains to interfere with the TLR4-mediated signaling triggered by *S. typhimurium* LPS. To appraise this ability, HEK-Blue hTLR4 cells were pretreated with different amounts (1, 10, and 100 ng/mL) of *E. pacifica* KMM 6172^T^ LPS or *E. vietnamensis* KMM 6221^T^ LPS, and then stimulated with *S. typhimurium* LPS (1 ng/mL) (Figure 8). NF-κB activation and IL-8 production were the read-outs also of these experiments. Interestingly, this study clearly showed that both *Echinicola* LPS significantly inhibited *S. typhimurium* LPS-dependent TLR4-mediated NF-κB activation and IL-8 production at all the concentrations tested (NF-κB activation, *E. pacifica* KMM 6172^T^ LPS vs. *S. typhimurium* LPS, *p* < 0.05 at 1 ng/mL; *p* < 0.01 at 10 ng/mL and *p* < 0.001 at 100 ng/mL, Figure 8a; *E. vietnamensis* KMM 6221^T^ LPS vs. *S. typhimurium* LPS, *p* < 0.05 at 10 ng/mL, Figure 8c; IL-8 production, *E. pacifica* KMM 6172^T^ LPS vs. *S. typhimurium* LPS, *p* < 0.001 at 100 ng/mL, Figure 8b; *E. vietnamensis* KMM 6221^T^ LPS vs. *S. typhimurium* LPS, *p* < 0.05 at 1 ng/mL, Figure 8d), with *E. pacifica* KMM 6172^T^ LPS displaying stronger antagonistic properties than *E. vietnamensis* KMM 6221^T^ LPS (Figure 8). Finally, we investigated whether *Echinicola* LPS could stimulate the TLR2-mediated signaling, as already observed for LPS of other Bacteroidetes such as *Porphyromonas gingivalis* and *Bacteroides vulgatus* [27,28]. Hence, HEK-Blue™ hTLR2 cells, obtained by co-transfection of the human TLR2 and SEAP genes into HEK293 cells, were stimulated with LPS from the two *Echinicola* strains as well as with LPS from *S. typhimurium* and analyzed as above for the HEK-Blue hTLR4 cells stimulation assay. In this case, *S. typhimurium* LPS and untreated cells were used as negative controls; on the other hand, the synthetic tri-acylated lipoprotein Pam3CSK4, which mimics the acylated amino terminus of bacterial lipoproteins, i.e., the natural TLR2 agonists, was used as a positive control. No significant TLR2 activation was observed with either *E. pacifica* KMM 6172^T^ LPS or *E. vietnamensis* KMM 6221^T^ LPS or *S. typhimurium* LPS (Figure S7).

## 4. Discussion

Low temperatures, high salt concentrations, and high hydrostatic pressures potently impact the structure of the membrane constituents of Gram-negative bacteria thriving in marine environments. As the portion of the LPS embedded in the Gram-negative outer membrane, the lipid A is one of the main bacterial components subjected to these selective pressures. As a consequence, lipid A from marine bacteria present several structural peculiarities diverging from those observed in the lipid A of their terrestrial counterparts [3]. Overall, most marine bacteria express lipid A molecules with a lower degree of acylation (tetra- and penta-acylated) than terrestrial bacteria, alongside with a tendency toward desaturation of the acyl chains, reduction in their length, and increase in their branching [3,29]. Moreover, the level of phosphorylation is also often lower than in terrestrial lipid A, with some marine species expressing mono- or even non-phosphorylated lipid A molecules [3,29]. 

**Figure 9:**
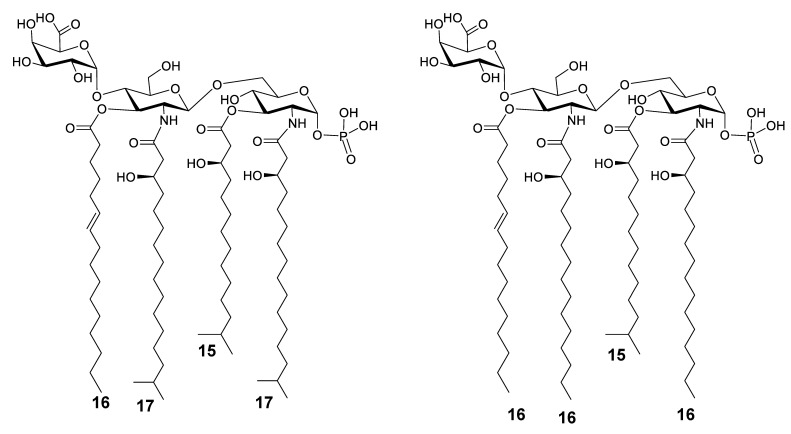
The proposed structure of the main lipid A species identified for *E. pacifica* KMM 6172^T^ (**a**) and *E. vietnamensis* KMM 6221^T^ (**b**) LPS via MALDI-TOF MS and MS/MS investigation in combination with compositional analyses.

Here, we showed that other unusual characteristics can be found in lipid A of some marine bacteria. Indeed, we demonstrated that the two sea *Echinicola* strains herein examined express a great variety of lipid A molecules, all united by the characteristic of being mono-phosphorylated and displaying only three or four acyl chains. In addition, a d-GalA was found linked to the nonreducing glucosamine of the lipid A disaccharide backbone. Briefly, the main species identified for *E. pacifica* KMM 6172^T^ was a tetra-acylated lipid A carrying two *N*-linked *i*17:0(3-OH), one primary *O*-linked 16:1, and one primary *O*-linked 15:0(3-OH), decorated by one phosphate on the reducing glucosamine and one d-GalA on the nonreducing glucosamine (Figure 9a); whereas, the main lipid A species of *E. vietnamensis* KMM 6221^T^ was mono-phosphorylated and decorated by d-GalA, *O*-linked 16:1 and 15:0(3-OH) as well, but in this case, the two *N*-linked acyl chains were 16:0(3-OH) (Figure 9b). Therefore, *E. pacifica* KMM 6172^T^ and *E. vietnamensis* KMM 6221^T^, as other marine bacteria, express hypo-acylated and hypo-phosphorylated lipid A molecules (Figure 9). Likewise, they also decorate their lipid A with unsaturated acyl chains, which are directly linked to the saccharide backbone as primary ester-linked moieties. This tendency of both *Echinicola* strains to increase the desaturation level of their membrane lipids can be seen as an ingenious strategy to increment the outer membrane fluidity by decreasing the lipid packing. Likely for the same purpose, *E. pacifica* and *E. vietnamensis* express lipid A that can be acylated also by short acyl moieties, such as *i*9:0(3-OH) or 10:0(3-OH), which establish lower interchain Van der Waals interactions than long-chain fatty acids, resulting in an additional contribute to preserving the membrane fluidity. Contextually, the occurrence of a d-GalA in place of a phosphate can be considered as an advantage for these bacteria; this because a glycosidic linkage is more resistant than an ester phosphate linkage, thereby, the presence of d-GalA may contribute to the membrane stability under hostile environmental conditions. More interestingly, a high content of odd numbered and branched acyl chains was also displayed for the two examined lipid A. In particular, similar to other bacteria of the Bacteroidetes phylum [27,28,30,31], both *Echinicola* lipid A are characterized by the presence of saturated, unsaturated, and hydroxylated acyl chains containing 15 and 17 carbon atoms, which are mostly present as branched structures. Curiously, this structural characteristic somehow reminisces the extremely unusual lipid A from the marine bacterium *Chryseobacterium scophtalmum* CIP 104199^T^, whose peculiarity is to consist in a single glucosamine residue, phosphorylated at C-1, and carrying one *i*17:0(3-OH) and one *i*15:0(3-OH) residues, as primary amide and ester substituents, respectively [32]; interestingly, this unusual structure, in turn, closely resembles the biosynthetic precursor of lipid A in *Escherichia coli*, the lipid X [33]. Certainly, the observed structural similitudes occurring between *C. scophtalmum* and *Echinicola* lipid A are extremely fascinating under an evolutionary and biosynthetic point of view, potentially providing additional information on the phenomenon of bacterial adaptation to the sea habitats. In addition, it should be mentioned that the occurrence of tri-acylated lipid A species raises important questions about their origin. At a first glance, it is tempting to correlate this to the occurrence in the *Echinicola* genome of the gene *lpxR* (Accession numbers for *E. pacifica* and *E. vietnamensis* are WP_018475561.1 and WP_157501590.1, respectively), identified first in *Salmonella enterica* serovar *Typhimurium* [34]. The enzyme (a 3′-*O*-deacylase) encoded by this gene is largely known for its calcium (Ca^2+^)-dependent capability to remove C-3′ acyl chains from lipid A of several *Salmonella* species as well as some other Gram-negative pathogens [34]. This is, in fact, considered advantageous during the infection process, allowing the bacterium to evade the innate immune response [5]. 

In this frame, therefore, deciphering the genes and the enzymes involved in the biosynthesis pathway of such extremely complex lipid A as well as the role of Ca^2+^, which is an important major cation in seawater, will be the object of future studies that promise results of unquestionable importance. 

From another perspective, the discovery of such unconventional structural features further proved the infinite and still unexplored chemical possibilities arising from bacteria experiencing marine environments, thus also becoming attractive from a chemical point of view. In this frame, it is even more interesting to disclose whether such unusual chemical structures are reflected in equally particular immunological properties. In light of this, by leveraging HEK-Blue cell lines stably expressing human TLR4, MD-2, and CD-14, we have highlighted the very weak capability of both *Echinicola* LPS to elicit a TLR4-mediated immune response while keeping the ability to bind the receptor. This interesting immunological behavior finds its reason to be in the hypo-acylated and hypo-phosphorylated chemical structure of the lipid A of both marine bacteria. In this frame, countless studies showed that hypo-acylated lipid A (namely lipid A species carrying less than six acyl chains) usually only poorly or do not activate the TLR4-mediated immune response [5]. Likewise, a reduced phosphorylation degree has been commonly associated to a scant immunoelicitation potency [5]. Even more fascinating has been the observation of the potent inhibitory activity shown by both *Echinicola* LPS towards the proinflammatory response induced by the *S. thypimurium* LPS on HEK-Blue^TM^ hTLR4 cells. This characteristic has been previously described for some other marine bacteria such as *Halobacteroides lacunaris* [11], *Cobetia pacifica* [10], as well as for the thermophilic bacterium *Thermomonas hydrothermalis* [35]. In this context, the present study further encourages the investigation of marine LPS as a never-ending source of natural molecules that can be potentially used to develop immunomodulatory compounds exploitable as therapeutics for inflammation-related pathologies.

## Figures and Tables

**Table 1:** Fatty acid content of the LPS isolated from the two *Echinicola* strains examined in the current study. Both strains displayed the same fatty acid composition and were both characterized by a disaccharide of d-glucosamine as the lipid A sugar backbone carrying an additional saccharidic moiety, i.e., a d-galacturonic acid. For the unsaturated acyl chains, the position of the double bond or the stereochemistry remain to be defined.

Fatty Acid Component
9:0 iso (3-OH) [*i*9:0(3-OH)]
10:0(3-OH)
14:0 15:1 iso (*i*15:1) 15:0 iso (*i*15:0) 15:0 anteiso (*a*15:0) 16:1 16:0 15:0 iso (3-OH) [*i*15:0(3-OH)] 15:0(3-OH) 17:0 iso (*i*17:0) 17:1 iso (*i*17:1) 16:0(3-OH) 17:0 iso 3-OH [*i*17:0(3-OH)]

**Table 2:** The main ion peaks observed in the MALDI-TOF MS spectra reported in Figure 1, the predicted masses, and the proposed interpretation of the substituting fatty acids, phosphate and HexA, on the lipid A backbone. The observed masses reported in the table are compared to the calculated monoisotopic mass (predicted mass, Da) of each ion based on the proposed lipid A structures.

***Echinicola pacifica* KMM 6172^T^**			
**Observed Ion Peaks (*m*/*z*)**	**Predicted Mass (Da)**	**Acyl Substitution**	**Proposed Composition**
1607.8	1608.0	Tetra-acyl	HexN^2^, *P*, HexA, [17:0(3-OH)]^2^ [15:0(3-OH)] (16:1)
1621.8	1622.0	Tetra-acyl	HexN^2^, *P*, HexA, [17:0(3-OH)]^2^ [15:0(3-OH)] (17:1)
1595.8	1596.0	Tetra-acyl	HexN^2^, *P*, HexA, [17:0(3-OH)]^2^ [15:0(3-OH)] (15:0)
1527.7	1527.9	Tetra-acyl	HexN^2^, *P*, HexA, [17:0(3-OH)]^2^ [15:0(3-OH)] [9:0(3-OH)]
1431.7	1432.0	Tetra-acyl	HexN^2^, *P*, [17:0(3-OH)]^2^ [15:0(3-OH)] (16:1)
1371.6	1371.8	Tri-acyl	HexN^2^, *P*, HexA, [17:0(3-OH)]^2^ [15:0(3-OH)]
***Echinicola vietnamensis* KMM 6221^T^**			
1579.8	1580.0	Tetra-acyl	HexN^2^, *P*, HexA, [16:0(3-OH)]^2^ [15:0(3-OH)] (16:1)
1513.7	1513.9	Tetra-acyl	HexN^2^, *P*, HexA, [16:0(3-OH)]^2^ [15:0(3-OH)] [10:0(3-OH)]
1403.8	1403.9	Tetra-acyl	HexN^2^, *P*, [16:0(3-OH)]^2^ [15:0(3-OH)] (16:1)
1343.6	1343.8	Tri-acyl	HexN^2^, *P*, HexA, [16:0(3-OH)]^2^ [15:0(3-OH)]

## Supplementary Materials

The following are available online at https://www.mdpi.com/article/10.3390/microorganisms9122552/s1, Figure S1: (a) Silver staining of SDS-PAGE of LPS from *Echinicola pacifica* KMM 6172^T^ (1) and *Echinicola vietnamensis* KMM 6221^T^ (2). In total, 8 μL of 1 mg/mL solution of both LPS were loaded in each lane of the gel. (b,c) Zoom of the GC-MS chromatogram profile recorded after methanolysis followed by acetylation of an aliquot of the isolated lipid A fraction from *E. pacifica* KMM 6172^T^ LPS (b) and *E. vietnamensis* KMM 6221^T^ LPS (c); Figure S2: Reflectron MALDI-TOF mass spectra of bacterial pellet of *E. pacifica* KMM 6172^T^ (a) and *E. vietnamensis* KMM 6221^T^ (b), both recorded in negative polarity. The lipid A species are labelled as Tri- and Tetra-Lip A indicating the degree of acylation; Figure S3: Negative-ion MALDI MS/MS spectrum of precursor ion at *m*/*z* 1595.8, chosen as an additional representative ion peak of the cluster assigned to tetra-acylated lipid A species from *E. pacifica* KMM 6172^T^. The assignment of main fragments is reported in the spectrum; Figure S4: Negative-ion MALDI MS/MS spectrum of precursor ion at *m*/*z* 1621.8, another representative ion peak of the cluster assigned to tetra-acylated lipid A species from *E. pacifica* KMM 6172^T^; Figure S5: Negative-ion MALDI MS/MS spectrum of precursor ion at *m*/*z* 1513.7, related to tetra-acylated lipid A species from *E. vietnamensis* KMM 6221^T^; Figure S6: Stimulation of HEK Blue^TM^ Null2^TM^ cells. SEAP levels (OD) (a) and IL-8 release (b) upon stimulation with LPS of *E. pacifica* KMM 6172^T^ (1, 10, 100 ng/mL) or *E. vietnamensis* KMM 6221^T^ LPS (1, 10, 100 ng/mL); *S. typhimurium* SH 2201 LPS was used as positive control; Figure S7: Stimulation of HEK Blue^TM^ hTLR2 cells. SEAP levels (OD) (a) and IL-8 release (pg/mL) (**b**) upon stimulation with LPS of *E. pacifica* KMM 6172^T^ (1, 10, 100 ng/mL) or *E. vietnamensis* KMM 6221^T^ LPS (1, 10, 100 ng/mL); *S. typhimurium* SH 2201 LPS or Pam3CSK4 (500 ng/mL) were used as positive and negative controls, respectively.
