# Supplementary material for: The Unusual Lipid A Structure and Immunoinhibitory Activity of LPS from Marine Bacteria Echinicola pacifica KMM 6172T and Echinicola vietnamensis KMM 6221T
Source: Microorganisms. 2021 Dec 10;9(12):2552. doi: 10.3390/microorganisms9122552 (PMC8707317; doi:10.3390/microorganisms9122552)
Supplement: Supplementary file 1 [file microorganisms-09-02552-s001.zip › microorganisms-1478576-supplementary.pdf]

Supplementary Material

The unusual lipid A structure and immunoinhibitory activity of LPS from marine bacteria *Echinicola pacifica* KMM 6172^T and *Echinicola vietnamensis* KMM 6221^T

Molly Dorothy Pither , Giuseppe Mantova, Elena Scaglione, Chiara Pagliuca, Roberta Colicchio, Mariateresa Vitiello, Oleg V. Chernikov, Kuo-Feng Hua, Maxim S. Kokoulin, Alba Silipo, Paola Salvatore, Antonio Molinaro, Flaviana Di Lorenzo

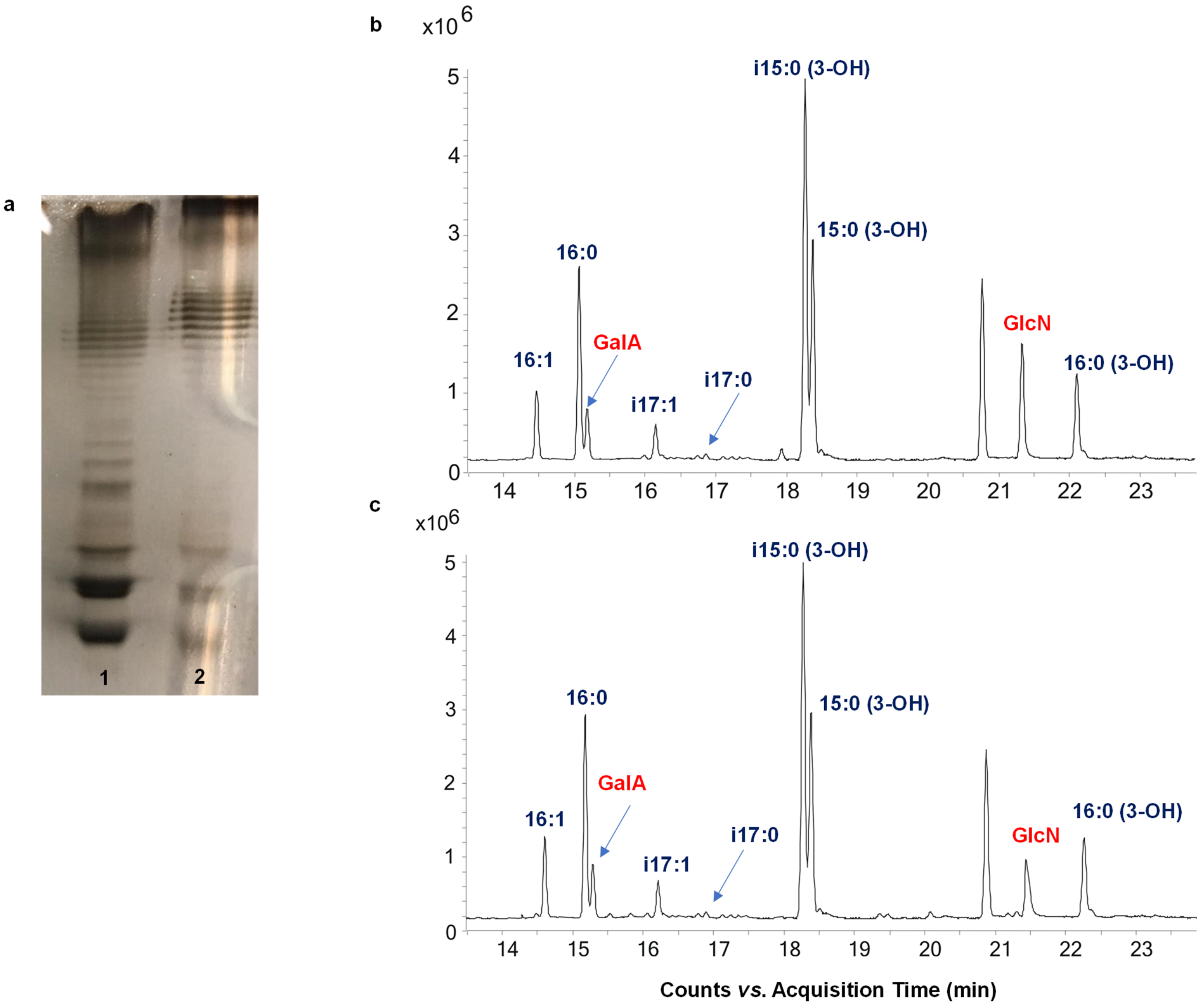

Figure S1. (**a**) Silver staining of SDS-PAGE of LPS from *Echinicola pacifica* KMM 6172^T (**1**) and *Echinicola vietnamensis* KMM 6221^T (**2**). 8 μL of 1 mg/mL solution of both LPS were loaded in each lane of the gel. (**b**,**c**) Zoom of the GC-MS chromatogram profile recorded after methanolysis followed by acetylation of an aliquot of the isolated lipid A fraction from *E. pacifica* KMM 6172^T LPS (**b**) and *E. vietnamensis* KMM 6221^T LPS (**c**). By this approach fatty acids are detected as methyl esters whereas monosaccharides composing the lipid A sugar backbone as acetylated methyl glycosides. The chromatograms clearly show the occurrence of galacturonic acid (GalA) and glucosamine (GlcN) as the sugar components of the two lipid A. Some of the fatty acids detected in this zoom section of the spectrum are also indicated.

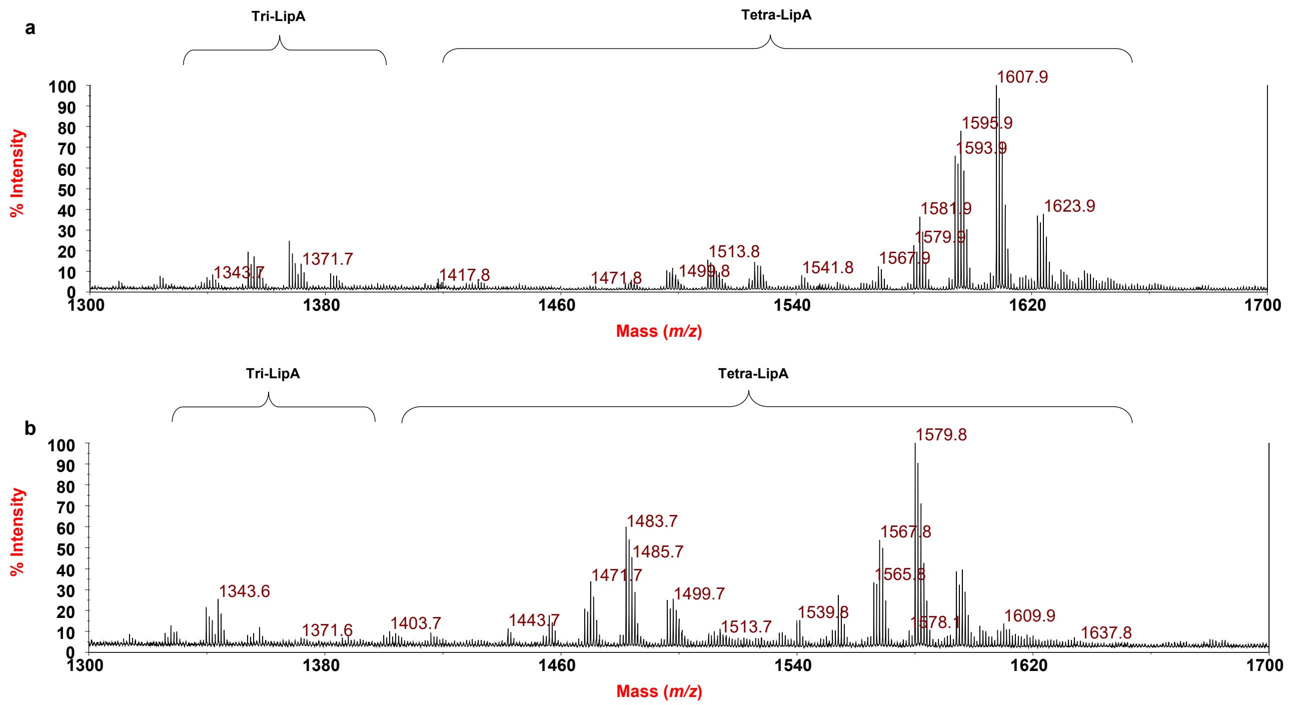

Figure S2. Reflectron MALDI-TOF mass spectra of bacterial pellet of *E. pacifica* KMM 6172^{T} (**a**) and *E. vietnamensis* KMM 6221^{T} (**b**), both recorded in negative polarity. The lipid A species are labelled as Tri- and Tetra-Lip A indicating the degree of acylation.

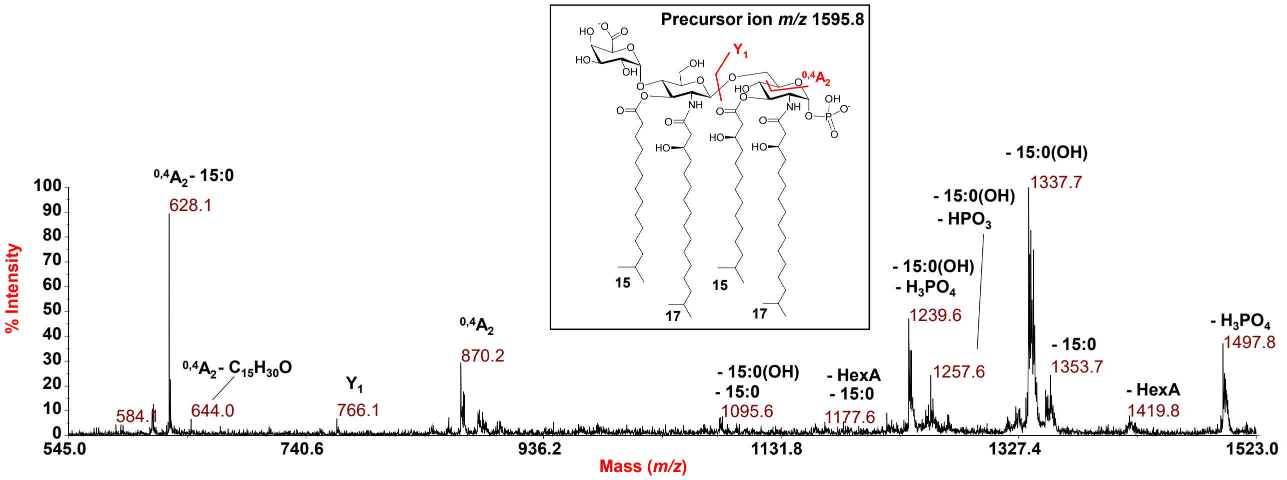

Figure S3. Negative-ion MALDI MS/MS spectrum of precursor ion at *m/z* 1595.8, chosen as an additional representative ion peak of the cluster assigned to tetra-acylated lipid A species from *E. pacifica* KMM 6172^T. The assignment of main fragments is reported in the spectrum. The proposed structure for the lipid A species is sketched in the inset, where the representation of the two 15-carbon atoms acyl chains in their *iso* form is tentative. As both the phosphate group and the HexA unit may hold the charge, the proposed structure has been sketched carrying two negative charges for information purposes only. The peak originating from the loss of $C_{15}H_{30}O$ (226 mass units) has been also reported in the spectrum.

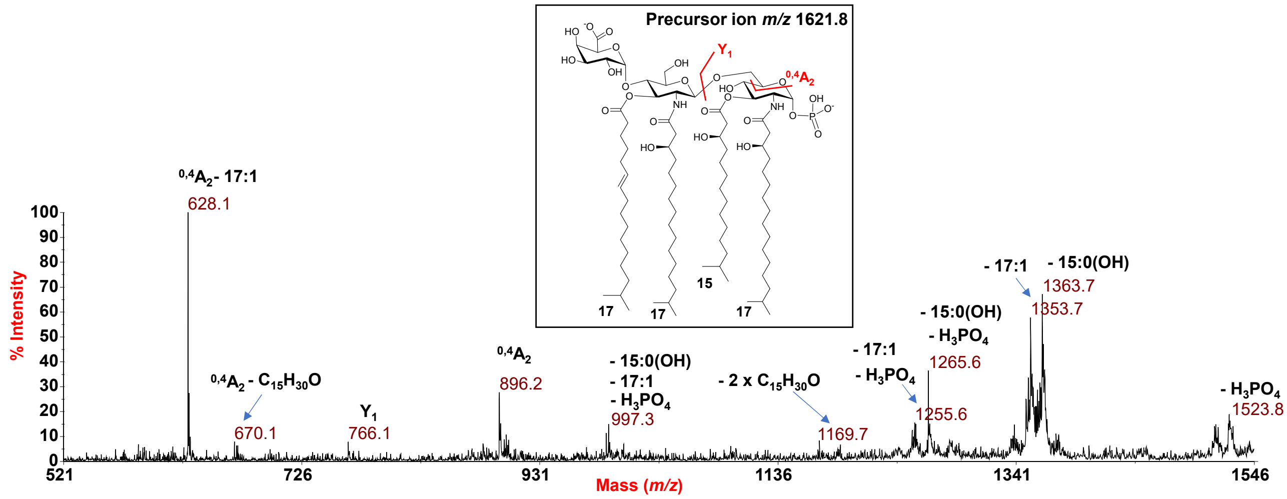

Figure S4. Negative-ion MALDI MS/MS spectrum of precursor ion at *m*/*z* 1621.8, another representative ion peak of the cluster assigned to tetra-acylated lipid A species from *E. pacifica* KMM 6172^{T}. The proposed structure for the lipid A species is reported in the inset, where the representation of the 15:0(3-OH) in its branching form is tentative and where the depiction of two negative charges is given for information purposes only.

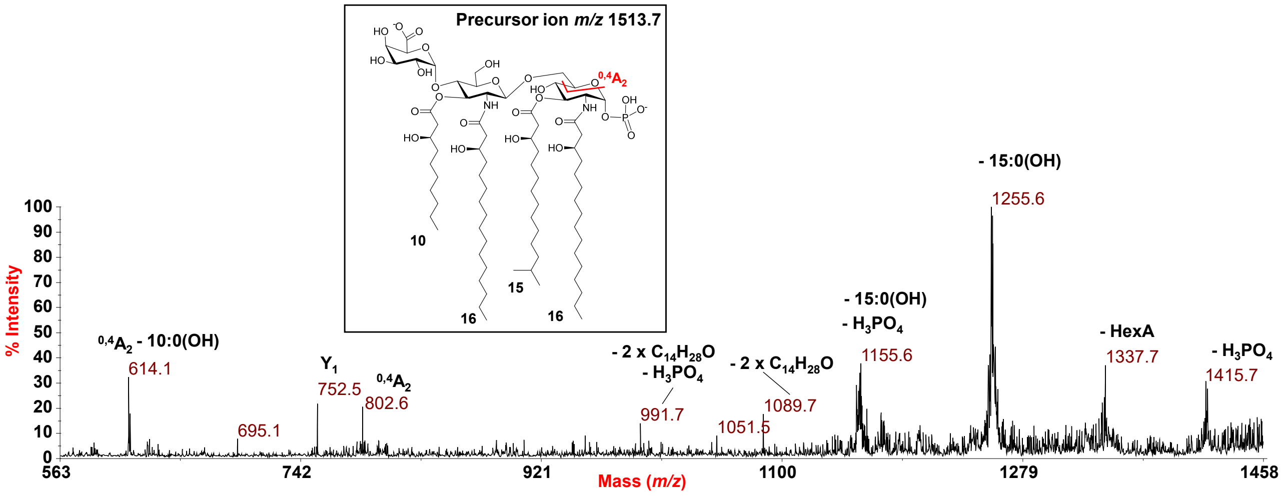

Figure S5. Negative-ion MALDI MS/MS spectrum of precursor ion at *m*/*z* 1513.7, related to tetra-acylated lipid A species from *E. vietnamensis* KMM 6221^T. The assignment of main fragments and the proposed structure for the lipid A species are reported in the spectrum. Also in this case, the representation of the 15:0(3-OH) in its branching form is tentative and the depiction of the structure carrying two negative charges is given for information purposes in order to underline that both sites of the molecule (i.e. the phosphate and the uronic acid) may hold the negative charge. Peaks originating from the loss of $C_{14}H_{28}O$ (212 mass units) have been indicated.

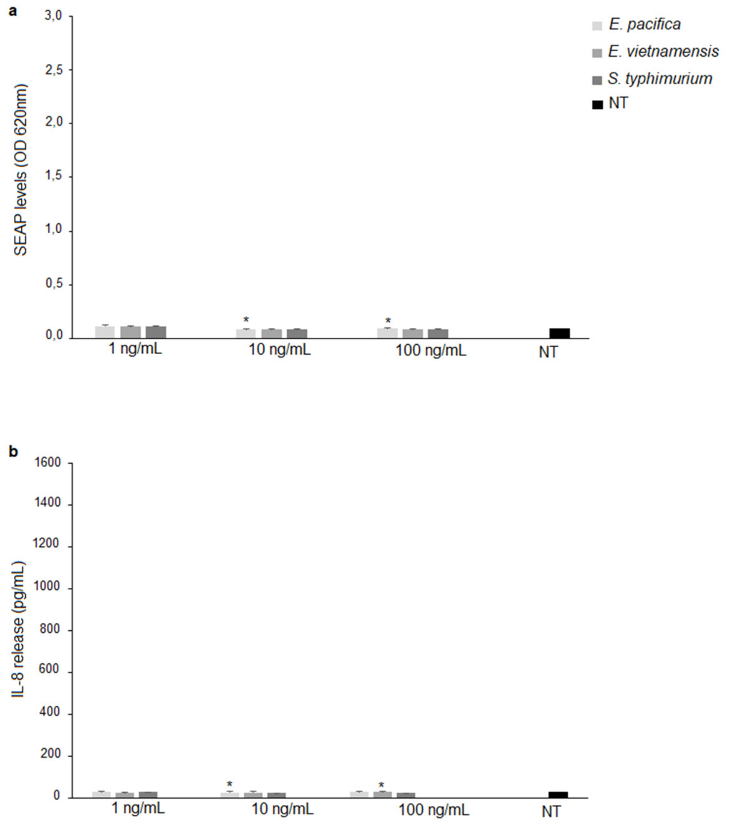

Figure S6. Stimulation of HEK Blue™ Null2™ cells. SEAP levels (OD) (**a**) and IL-8 release (**b**) upon stimulation with LPS of *E. pacifica* KMM 6172^T (1, 10, 100 ng/mL) or *E. vietnamensis* KMM 6221^T LPS (1, 10, 100 ng/mL); *S. typhimurium* SH 2201 LPS was used as positive control. Significant differences between *E. pacifica* KMM 6172^T LPS or *E. vietnamensis* KMM 6221^T LPS and *S. typhimurium* LPS values are indicated. *$p < 0.05$ by Student's *t*-test. NT, not treated cells. Data are expressed as mean ± SD of three independent experiments in triplicate.

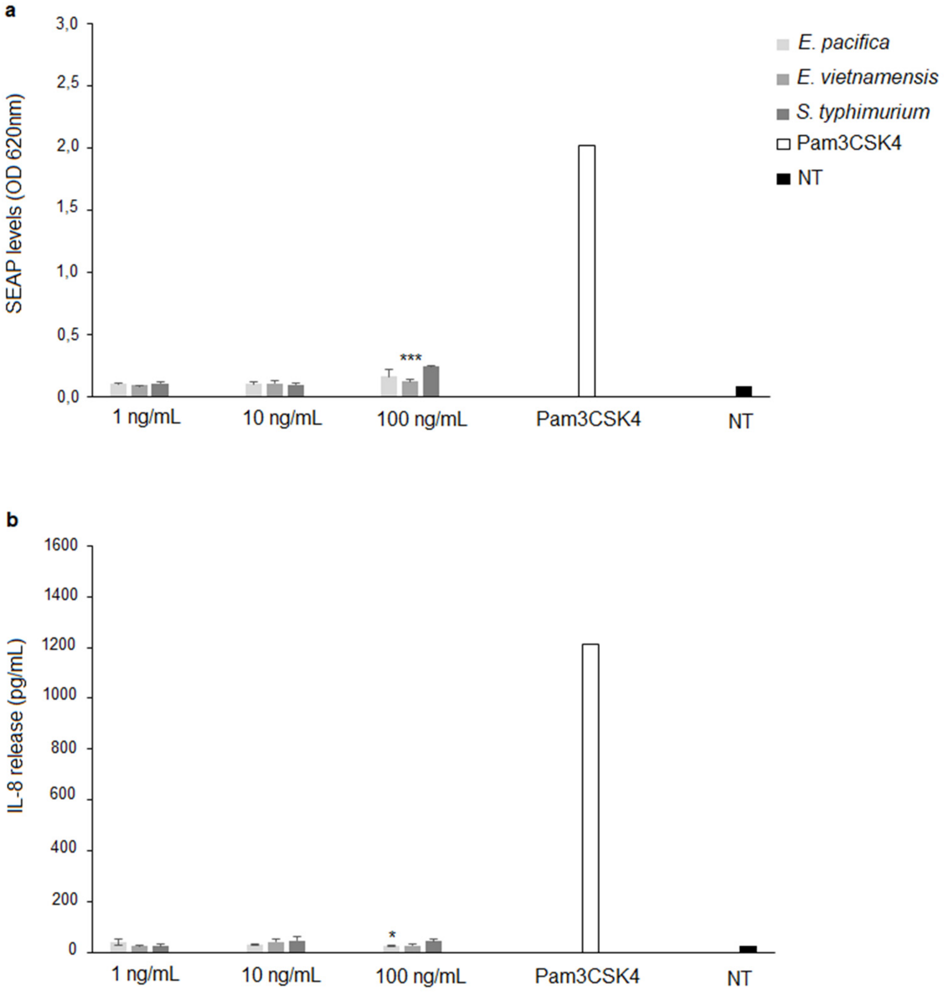

Figure S7. Stimulation of HEK Blue™ hTLR2 cells. SEAP levels (OD) (**a**) and IL-8 release (pg/mL) (**b**) upon stimulation with LPS of *E. pacifica* KMM 6172^T (1, 10, 100 ng/mL) or *E. vietnamensis* KMM 6221^T LPS (1, 10, 100 ng/mL); *S. typhimurium* SH 2201 LPS or Pam3CSK4 (500 ng/mL) were used as positive and negative controls, respectively. Significant differences between *E. pacifica* KMM 6172^T LPS or *E. vietnamensis* KMM 6221^T LPS and the corresponding *S. typhimurium* LPS values are indicated. *p < 0.05, ***p < 0.001 by Student's *t*-test. NT, not treated cells. Data are expressed as mean ± SD of three independent experiments in triplicate.
